# Cancer and fertility preservation: international recommendations from an expert meeting

**DOI:** 10.1186/s12916-015-0545-7

**Published:** 2016-01-04

**Authors:** Matteo Lambertini, Lucia Del Mastro, Maria C. Pescio, Claus Y. Andersen, Hatem A. Azim, Fedro A. Peccatori, Mauro Costa, Alberto Revelli, Francesca Salvagno, Alessandra Gennari, Filippo M. Ubaldi, Giovanni B. La Sala, Cristofaro De Stefano, W. Hamish Wallace, Ann H. Partridge, Paola Anserini

**Affiliations:** Department of Medical Oncology, U.O. Oncologia Medica 2, IRCCS AOU San Martino – IST, Genoa, Italy; Department of Medical Oncology, U.O. Sviluppo Terapie Innovative, IRCCS AOU San Martino – IST, Genoa, Italy; Physiopathology of Human Reproduction, IRCCS AOU San Martino – IST, Genoa, Italy; Laboratory of Reproductive Biology, Section 5712, Juliane Marie Centre for Women, Children and Reproduction, University Hospital of Copenhagen, Copenhagen, Denmark; BrEAST Data Centre, Department of Medicine, Institut Jules Bordet, Université Libre de Bruxelles, Brussels, Belgium; Fertility and Procreation Unit, Gynecologic Oncology Department, European Institute of Oncology, Milan, Italy; Reproductive Medicine Department, International Evangelic Hospital, Genoa, Italy; Physiopathology of Reproduction and In Vitro Fertilization Unit, S. Anna Hospital, University of Turin, Turin, Italy; Department of Medical Oncology, E.O. Galliera, Genoa, Italy; GENERA Centre for Reproductive Medicine, Clinica Valle Giulia, Rome, Italy; Obstetric and Gynecology Department, Azienda Ospedaliera Arcispedale S. Maria Nuova-IRCCS, University of Modena and Reggio Emilia, Reggio Emilia, Italy; Children and Women Health Department, Physiopathology of Human Reproduction Unit, “San Giuseppe Moscati” Hospital, Avellino, Italy; Department of Haematology/Oncology, Royal Hospital for Sick Children, and Department of Child Life and Health, University of Edinburgh, Edinburgh, UK; Department of Medical Oncology, Dana-Farber Cancer Institute, Boston, MA USA

**Keywords:** Fertility preservation, cancer patients, survivorship issues, sperm cryopreservation, embryo cryopreservation, oocyte cryopreservation, ovarian tissue cryopreservation, luteinizing hormone-releasing hormone analogs

## Abstract

In the last years, thanks to the improvement in the prognosis of cancer patients, a growing attention has been given to the fertility issues. International guidelines on fertility preservation in cancer patients recommend that physicians discuss, as early as possible, with all patients of reproductive age their risk of infertility from the disease and/or treatment and their interest in having children after cancer, and help with informed fertility preservation decisions. As recommended by the American Society of Clinical Oncology and the European Society for Medical Oncology, sperm cryopreservation and embryo/oocyte cryopreservation are standard strategies for fertility preservations in male and female patients, respectively; other strategies (e.g. pharmacological protection of the gonads and gonadal tissue cryopreservation) are considered experimental techniques. However, since then, new data have become available, and several issues in this field are still controversial and should be addressed by both patients and their treating physicians.

In April 2015, physicians with expertise in the field of fertility preservation in cancer patients from several European countries were invited in Genova (Italy) to participate in a workshop on the topic of “cancer and fertility preservation”. A total of ten controversial issues were discussed at the conference. Experts were asked to present an up-to-date review of the literature published on these topics and the presentation of own unpublished data was encouraged. On the basis of the data presented, as well as the expertise of the invited speakers, a total of ten recommendations were discussed and prepared with the aim to help physicians in counseling their young patients interested in fertility preservation.

Although there is a great interest in this field, due to the lack of large prospective cohort studies and randomized trials on these topics, the level of evidence is not higher than 3 for most of the recommendations highlighting the need of further research efforts in many areas of this field. The participation to the ongoing registries and prospective studies is crucial to acquire more robust information in order to provide evidence-based recommendations.

## Background

Although cancer incidence increases with age and peaks after the age of 50, thousands of young women and men are diagnosed with cancer every year [1]. Modern anticancer treatments have led to significant reduction in mortality, but also to an increase in unwanted side effects such as reduced fertility. Combined with an increased age for childbearing [2], a higher number of cancer survivors require fertility preservation to complete their families.

The threat or experience of treatment-related infertility can lead to psychological distress [3], and many patients are interested in maintaining fertility and future reproductive function at the time of cancer diagnosis [4, 5]. Moreover, fertility concerns may also substantially affect their treatment decisions [6–8]. Thus, great attention to fertility issues is warranted at the time of cancer diagnosis.

International guidelines recommend that physicians discuss, as early as possible, with all patients of reproductive age their risk of infertility from the disease and/or treatment and their interest in having children after cancer, and help with informed fertility preservation decisions [9–11]. As recommended by the American Society of Clinical Oncology (ASCO) and the European Society for Medical Oncology (ESMO), sperm cryopreservation and embryo/oocyte cryopreservation are standard strategies for fertility preservation in male and female patients, respectively [10, 11]. Other strategies (e.g. pharmacological protection of the gonads and gonadal tissue cryopreservation) are still generally considered experimental techniques [10, 11]. However, new data in this field have become available over the past few years for consideration. Furthermore, several issues remain controversial with regard to the safety and efficacy of fertility preservation strategies in cancer survivors. In the present manuscript, we summarize and discuss the up-to-date knowledge on ten controversial topics in the field of fertility preservation in young patients with cancer.

## Methods

In April 2015, physicians with expertise in the field of fertility preservation in cancer patients from several European countries were invited in Genova (Italy) to participate in a workshop on the topic of “cancer and fertility preservation”. The invited experts represented different disciplines related to the topic including oncologists, hematologists, gynecologists, fertility specialists.

A total of ten controversial issues were focused on at the conference. Experts were asked to present an up-to-date review of the literature on these topics. In addition, the presentation of own unpublished data when available was encouraged.

On the basis of the data presented, as well as the expertise of the invited speakers, recommendations surrounding each topic were discussed and prepared with the aim to help physicians in counseling their young cancer patients interested in fertility preservation. To gain a United States (US) perspective, the recommendations were discussed and reviewed by Dr. Ann H. Partridge, from the Dana-Farber Cancer Institute, Boston (MA).

To evaluate the levels of evidence and grades of recommendation, the grading system commonly used in oncology was chosen (Table 1) [11].

**Table 1:** Levels of evidence and grades of recommendation (according to the ESMO Clinical Practice Guidelines for fertility preservation in cancer patients [11])

Levels of evidence	
I	Evidence from at least one large randomized, controlled trial of good methodological quality (low potential for bias) or meta-analyses of well-conducted randomized trials without heterogeneity
II	Small randomized trials or large randomized trials with a suspicion of bias (lower methodological quality) or meta-analyses of such trials or of trials with demonstrated heterogeneity
III	Prospective cohort studies
IV	Retrospective cohort studies or case–control studies
V	Studies without control group, case reports, experts opinions
Grade of recommendation	
A	Strong evidence for efficacy with a substantial clinical benefit, strongly recommended
B	Strong or moderate evidence for efficacy but with a limited clinical benefit, generally recommended
C	Insufficient evidence for efficacy or benefit does not outweigh the risk or the disadvantages (adverse events, costs, etc.), optional
D	Moderate evidence against efficacy or for adverse outcome, generally not recommended
E	Strong evidence against efficacy or for adverse outcome, never recommended

### Controversial issues in fertility preservation in cancer patients

#### Is there an increased risk of developing breast cancer in women treated with ovarian stimulating drugs for infertility?

Elevated blood level of endogenous estrogen are known to be associated with an increased risk of breast cancer [12–14]. A large meta-analysis including more than 50 observational studies for a total of 160,000 women, demonstrated a higher risk of having breast cancer (relative risk [RR] = 1.35; 95 % confidence intervals [CI] 1.21-1.49) for users of hormone-replacement therapy (HRT) for 5 years or longer [15].

For these reasons, some concerns exist on the safety of using ovarian stimulating drugs. However, unlike HRT, characterized by a long lasting exposure to low estrogen levels and more robust data on the subsequent risk of breast cancer with this approach [15], the use of ovarian stimulating drugs in subfertile patients is associated with significantly higher circulating estrogen levels for transient and shorter periods but with conflicting results on the risk of breast neoplasms [16, 17].

Recently, a systematic review and meta-analysis of cohort studies evaluated the association between hormonal infertility treatments and risk of developing breast cancer [18]. A total of 20 studies with 207,914 women exposed to hormonal treatments for infertility and 2,347 incident cases of breast cancer were included. Overall, no increased risk was detected with the use of hormonal treatments for infertility (summary RR [SRR] = 1.05; 95 % CI 0.96-1.14) but a significant heterogeneity among studies was detected (I^2^ = 59 %; *P* = 0.001) [18]. In subgroup analyses, when considering only the seven studies with the in vitro fertilization (IVF) procedure, no increase in breast cancer risk was detected (SRR = 0.96; 95 % CI 0.81-1.14). On the contrary, a moderately increased breast cancer risk was observed in the three studies where women were treated outside IVF protocols (SRR = 1.26; 95 % CI 1.06-1.50): however, to note, in these studies patients were enrolled before 1980. Overall, the meta-analysis did not support the hypothesis that hormonal treatments for infertility are associated with an increased breast cancer risk [18].

Recently, a large US cohort study reported reassuring results about the long-term effects of ovarian stimulating drugs with clomiphene or gonadotropins [19]. After a median follow up of 30 years, out of 9,892 women evaluated for infertility, 749 developed breast cancer. Ever use of clomiphene citrate was not associated with risk (hazard ratio [HR] = 1.05; 95 % CI 0.90-1.22). However, a significantly elevated risk was observed for patients who received both a high cumulative dose (i.e. ≥ 2251 mg) and multiple cycles (i.e. ≥ 6 cycles), with an HR of 1.27 (95 % CI 1.02-1.59). Ever use of gonadotropins was not significantly associated with the risk of developing breast cancer (HR = 1.14; 95 % CI 0.89-1.44), with no trends according to dosage, number of cycles, or age at first use [19].

Despite these encouraging findings for infertile women who wish to undergo IVF procedures, caution is needed because it is difficult to assess the risk of breast cancer in this setting. In fact, several factors (e.g. pregnancy, infertility itself and the use of different therapeutic protocols) might impact study results due to their direct or indirect effect on the risk of developing breast cancer [18]. Moreover, the evidence derived from observational studies have possible biases, including selection bias and ascertainment bias [18].

##### Recommendation 1

Ovarian stimulating drugs with standard treatment protocols may be administered in subfertile/infertile women without increasing the risk of developing breast cancer (III, B). The long-term use of clomiphene outside the current limited indications (i.e. first-line therapy of WHO Group II anovulatory infertility) should be discouraged because of a possible increase in breast cancer risk (III, B).

#### Should pregnancy after cancer be considered safe?

A considerable proportion of cancer patients of childbearing age (approximately 50 %) desire pregnancy at the time of cancer diagnosis [20]. However, cancer survivors have lower fecundity rates than the general population. A large population-based matched cohort study showed that despite an overall lower pregnancy rate in cancer survivors (23 % of men and 13 % of female) than in controls from the general population (32 % of men and 22 % of female), male survivors initiated pregnancies in a higher proportion (HR = 0.74; 95 % CI 0.71-0.78) than female survivors (HR = 0.61; 95 % CI 0.58-0.64) [21]. Patients with malignant melanoma or thyroid cancer showed a similar pregnancy rate as compared to the controls: a possible reason is that the treatments required for these diseases when caught at early stages have no major negative impact on patients’ fertility [21]. Survivors of ovarian cancer, testicular cancer, and Hodgkin lymphoma diagnosed in men, showed increasing pregnancy rates over time [21]. On the other hand, survivors of leukemia, cervical cancer or breast tumors showed the lowest rates for subsequent pregnancies [21].

Breast cancer patients have the lowest pregnancy rate among cancer survivors, with an overall 67 % reduction in the chance of having babies after cancer treatment as compared to the general population [21]. This observation reflects not only the damage to ovarian reserve due to the gonadotoxic treatments required, but also patient and provider concerns related to a possible negative impact of pregnancy on the evolution of breast cancer, being a hormonally driven disease. However, available data suggest that pregnancy after breast cancer does not negatively impact patients’ prognosis, irrespectively of the hormone receptor status of the tumor. A meta-analysis of 14 retrospective control-matched studies showed that breast cancer patients who became pregnant following diagnosis and treatment had a 41 % reduced risk of death compared to women who did not get pregnant (pooled RR [PRR] = 0.59; 95 % CI 0.50-0.70) [22]. While these results could be partially confounded by selection bias or the healthy mother effect, and lack of information in hormone receptor positive patients, a more recent multicenter retrospective cohort study adjusting for this effect confirmed safety of pregnancy after breast cancer even in patients with endocrine-sensitive disease [23]. This study showed no difference in disease-free survival (DFS) between pregnant and non-pregnant patients in the estrogen-receptor positive group (HR = 0.91; 95 % CI 0.67-1.24), nor in the estrogen-receptor negative cohort (HR = 0.75; 95 % CI 0.51-1.08) [23]. Importantly, no impact of abortion on patient outcome was observed either [23].

In addition, the possible occurrence of congenital abnormalities and the potential obstetric and birth complications are two main concerns for young cancer survivors who want to reproduce after anticancer treatment. In general, reseach has revealed that the neonatal outcomes in both male and female survivors are not different from those of the general population [24–29]. However, high induced abortion rates have been observed in patients who became pregnant after breast cancer diagnosis reaching as high as 30 % [23, 30, 31]. Moreover, in breast cancer survivors, a higher incidence of birth complications (i.e. caesarean section, preterm birth, babies with low birth weight) was observed in patients as compared to controls [25]. Women undergoing pelvic irradiation might experience uterine damage with a possible increased risk of miscarriage, preterm birth, and low birth weight [32]. For all these reasons, a close monitoring of pregnancy in such patients is recommended [33].

To date, pregnancy after cancer should be considered safe and not be discouraged in general [11]. However, it is not clear yet the ideal interval to wait between the end of anticancer treatments and conception. Two main interval issues should be considered: to wait until the patient is at lower risk of relapse, and to wait until the anticancer therapy is out of a patient’s system (i.e. up to 3–6 months following the last administered dose) [11]. According to expert opinion, the timing should be “personalized” taking into account age and ovarian reserve of the patient, previous treatments and time of their completion, and individual risk of relapse [11]. In breast cancer patients with hormone receptor-positive disease, the need for adjuvant endocrine therapy is another important issue to be considered: its use for up to 5–10 years can lead to a natural decline in ovarian reserve due to age, thus hindering the chances of future pregnancies. An international prospective study with the aim to evaluate the feasibility and safety of a temporary interruption of endocrine therapy to allow pregnancy is ongoing (the POSITIVE study) [34].

##### Recommendation 2

Pregnancy in cancer survivors, after adequate treatment and follow up, should not be discouraged, including among patients with endocrine-sensitive breast cancer (III, A).

#### Should all patients be referred to a fertility unit before initiating anticancer treatments?

Anticancer treatments (surgery, radiotherapy, cytotoxic chemotherapy and endocrine therapy) may affect male and female fertility transiently or permanently [9]. Surgery can have a direct impact on patients’ fertility causing anatomic or vascular problems (e.g. retrograde ejaculation or impaired ejaculation, changes to the uterus, cervix or vagina) [9]. Chemotherapy and radiotherapy have a direct gonadotoxic effect by destroying ovarian follicles that constitute woman’s ovarian reserve [35–37] and by compromising sperm number, motility, morphology, and DNA integrity in male patients [9]. Finally, endocrine treatments used in breast cancer patients have both a direct and indirect effect on fertility and ovarian function: the direct effect, occurring during treatment only, is due to an impairment in ovulatory and endometrial functions, while the indirect effect is associated with the delay to conception that allows ovarian aging.

As recommended by major international guidelines on fertility preservation in cancer patients, clinicians should discuss with their patients the potential impact of anticancer treatment on fertility as early as possible and help with fertility preservation decisions among at-risk survivors interested in having children after cancer [10, 11, 38].

However, the rate of treatment-related infertility is variable and depends on several factors: type of cancer, age of the patient, history of previous treatment for infertility and comorbidities, type and dose of the chemotherapy regimen used, method of administration (oral versus intravenous), size and location of the radiation field and its dose, need of adjuvant endocrine therapy (Table 2) [9, 39]. Specifically, type of treatment and patients’ age are the most important factors to be taken into account when counseling the patients [40].

**Table 2:** Risk of treatment-related infertility with the main anticancer therapies (modified from the original [9])

Degree of risk	Type of anticancer treatment	
	Women	Men
High risk (>80 % risk of permanent amenorrhea in women; prolonged azoospermia in men)	-HSC transplantation with cyclophosphamide/TBI or cyclophosphamide/busulfan -External beam radiation to a field that includes the ovaries -CMF, CEF, CAF, TAC x 6 cycles in women ≥ 40 years	-Radiation > 2.5 Gy to testis -Chlorambucil (1.4 g/m2) -Cyclophosphamide (19 g/m2) -Procarbazine (4 g/m2) -Melphalan (140 mg/m2) -Cisplatin (500 mg/m2) -BCNU (1 g/m2) and CCNU (500 mg/m2)
Intermediate risk (40 % - 60 % risk of permanent amenorrhea in women; likelihood of azoospermia in men especially when given with other sterilizing agents)	-BEACOPP -CMF, CEF, CAF, TAC x 6 cycles in women age 30–39 -AC x 4 cycles in women ≥ 40 years -AC or EC x 4 → Taxanes	-Busulfan (600 mg/kg) -Ifosfamide (42 g/m2) -BCNU (300 mg/m2) -Nitrogen mustard -Actinomycin D
Low risk (<20 % risk of permanent amenorrhea in women; only temporary reductions in sperm counts in men especially when not given with other sterilizing agents)	-ABVD in women ≥ 32 years -CHOP x 4–6 cycles -CVP -AML therapy (anthracycline/cytarabine) -ALL therapy (multi-agent) -CMF, CEF, CAF, TAC x 6 cycles in women ≤ 30 years -AC x 4 cycles in women ≤ 40 years	-Carboplatin (2 g/m2) -Doxorubicin (770 mg/m2) -Thiotepa (400 mg/m2) -Cytosine arabinoside (1 g/m2) -Vinblastine (50 g/m2) -Vincristine (8 g/m2)
Very low or no risk (risk of permanent amenorrhea in women; temporary reductions in sperm count in men but additive effects are possible)	-ABVD in women < 32 years -Methotrexate -Fluorouracil -Vincristine -Tamoxifen	-Amsacrine -Bleomycin -Dacarbazine -Daunorubicin -Epirubicin -Etoposide -Fludarabine -Fluorouracil −6-mercaptopurine -Methotrexate -Mitoxantrone, -Thioguanine -Prednisone -Interferon-α
Unknown risk (risk of permanent amenorrhea in women; effect on sperm production in men)	-Monoclonal antibodies (trastuzumab, bevacizumab, cetuximab) -Tyrosine kinase inhibitors (erlotinib, imatinib)	-Oxaliplatin -Irinotecan -Monoclonal antibodies (trastuzumab, bevacizumab, cetuximab) -Tyrosine kinase inhibitors (erlotinib, imatinib) -Taxanes

*HSC* hematopoietic stem cell, *TBI* total body irradiation, *CMF* cyclophosphamide, methotrexate, fluorouracil, *CEF* cyclophosphamide, epirubicin, fluorouracil, *CAF* cyclophosphamide, doxorubicin, fluorouracil, *TAC* docetaxel, doxorubicin, cyclophosphamide,* BEACOPP* doxorubicin, belomycin, vincristine, etoposide, cyclophosphamide, procarbazine, *BCNU* carmustine, *CCNU* lomustine, *AC* doxorubicin, cyclophosphamide, *EC* epirubicin, cyclophosphamide, *ABVD* doxorubicin, bleomycin, vinblastin, dacarbazine, *CHOP* cyclophosphamide, doxorubicin, vincristine, prednisone, *CVP* cyclophosphamide, vincristine, prednisone, *AML* acute myeloid leukemia, *ALL* acute lymphocitic leukemia

Onco-fertility counseling should be individualized, discussing both the absolute benefits of the proposed anticancer treatment (e.g. adjuvant chemotherapy or long duration of endocrine therapy in young breast cancer patients at low risk of recurrence) and the risk of infertility for each individual (based on patient-related factors [age, comorbidities, ovarian reserve in women [41]] and the sterilizing potential of the treatment proposed). In some situations the risk of treatment-related infertility can be difficult to estimate due to limited available data; however, providers should not overestimate the risk of treatment-related infertility for cancer patients and some of them (e.g. very young patients undergoing treatment at low risk of infertility) can be reassured that they will not likely require the help of a fertility clinic after cancer treatment [42]. On the other hand, the perception of a high risk for infertility is individual and the patients’ own wishes should also be taken into account. Further, to note that even with regimens associated with low risk of gonadotoxicity (e.g. doxorubicin,bleomycin,vinblastine,dacarbazine [ABVD] in Hodgkin lymphoma patients), nonetheless ovarian reserve is reduced and fertility may be impaired, particularly when pregnancy is delayed [43, 44]. Moreover, some patients may relapse and subsequently become candidates for different types of chemotherapy and at higher doses with a consequent negative impact on their gonadal function.

##### Recommendation 3

All patients with potential interest in keeping their fertility should be referred to fertility unit for adequate determination of risk of infertility, chances of future conception and how to proactively preserve it (V, A). However, some cancer patients will not require the help of a fertility clinic after cancer treatment (V, B). Since several patient- and treatment-related factors are associated with the risk of developing infertility, the oncofertility counseling should be tailored to the individual patient (V, A).

#### Are gamete and embryo cryopreservation strategies accepted by young cancer patients, when available and accessible?

Despite a growing amount of evidence suggesting that fertility issues are of great importance for young cancer patients, how many are counseled and offered these procedures is not well documented. In men, sperm cryopreservation is an effective strategy for fertility preservation: it does not require major delays in treatment initiation and can be easily performed in many centers [45]. In women, access to standard strategies (oocytes/embryos cryopreservation) may be more difficult because of urgent need for treatment, and lack of a well-organized interaction between oncology and fertility units.

The percentage of women who choose to undergo the available fertility preservation options after fertility counseling varies from 2 % to over 50 % [46, 47]. In a recent large study conducted in breast cancer patients, only 10 % of women took active steps to reduce their chance of infertility (7 % underwent embryo cryopreservation, 1 % oocyte cryopreservation and 3 % accepted the administration of luteinizing hormone-releasing hormone analogs [LHRHa]); however, an increasing trend over time in the proportion of patients who pursued fertility preservation strategies was observed (from 5 % in 2006 to 15 % in 2012) [7].

In a recent prospective study conducted in 7 large Italian Institutions, the rate of application of cryopreservation techniques was evaluated in male and female cancer patients who underwent onco-fertility counseling over a period of 2 years (unpublished observations). Out of 510 men, 507 (99.4 %) cryopreserved at least one semen sample, and 88 (17.4 %) patients underwent two or more sperm collections. Out of 491 women, 132 (26.8 %) were considered not eligible for cryopreservation strategies (i.e. due to need to start chemotherapy immediately, inadequate ovarian reserve, high risk of complication or use of treatments with a low risk of gonadotoxicity). Among eligible patients, 66.6 % underwent cryopreservation techniques to preserve fertility (Table 3). The lack of discussion of fertility issues between patients and physicians [47–49], and inadequate access to the strategies, are possible explanations of these findings [50, 51].

**Table 3:** The choices regarding fertility preservation of female cancer patients enrolled in the Italian study

Type of cancer *n* (%)	Eligible for cryopreservation strategies *n* (%)	Declined cryopreservation strategies *n* (%)	Accepted oocyte cryopreservation *n* (%)	Accepted ovarian tissue cryopreservation *n* (%)
Breast 281 (57.2)	186 (66.2)	76 (40.9)	103 (55.4)	7 (3.8)
Hematologic disease 123 (25.1)	98 (79.7)	27 (27.6)	50 (51.0)	21 (21.4)
Others 87 (17.7)	75 (86.2)	17 (22.7)	38 (50.7)	20 (26.7)
Total 491 (100)	359 (73.1)	120 (33.4)	191 (53.2)	48 (13.4)

Greater effort is needed to improve both the communication between patients and physicians about fertility risks and preservation options, and the collaboration among oncologists and fertility specialists to give patients the opportunity to undergo well-timed and complete reproductive counseling. Further research is needed to better understand the preferences of patients among the available strategies for fertility preservation: this information would have great importance from a public health perspective and for resource allocation standpoint. Moreover, a better understanding of the factors that influence patients’ choice would help physicians to improve the quality of their fertility counseling.

##### Recommendation 4

In men, sperm cryopreservation is an easily accessible and widely available option in more than 95 % of patients and should be encouraged for those who want to preserve fertility (III, A). On the contrary, from 2 % to 65 % of women undergo one of the available cryopreservation options: oncologists should discuss with them the fertility issues and secure proper counseling in appropriate centers prior to cancer treatment (IV, A).

#### Which are the results and the safety of assisted reproduction technologies (ART) in male patients previously treated with chemotherapy?

Anticancer treatments (i.e. chemotherapy and radiotherapy) can damage the germinal epithelium in men resulting in oligozoospermia or azoospermia: in fact, a large proportion of patients treated for cancer have lower sperm concentrations than matched controls [9]. Currently, no strategies for medical protection of the germinal epithelium are available. Further, prepubertal age cannot be considered a protective factor from gonadotoxic insults. Data suggest that cancer itself can influence spermatogenesis [52]. However, no correlation between semen alterations and cancer stage or associated symptoms has been detected [53, 54].

Sperm cryopreservation before gonadotoxic therapies is the standard strategy for fertility preservation in adult men [10, 11]. A large proportion of treated patients maintains or regains a level of spermatogenesis adequate to obtain spontaneous conception [55]. However, male cancer survivors who don’t recover spermatogenesis nor had their semen cryopreserved before cytotoxic therapy may need ART, and specifically intracytoplasmatic sperm injection (ICSI), in order to reproduce. Therefore, when considering the safety of conception after radiotherapy and/or chemotherapy, there are three different scenarios: a) spontaneous conception in patients with continued post radiotherapy/chemotherapy spermatogenesis; b) ART conception with normal or abnormal cryopreserved sperm; c) ART conception with post radiotherapy/chemotherapy residual spermatogenesis (ICSI particularly).

The limited available data on fatherhood after cancer are related to offspring from spontaneous conceptions or from IVF/ICSI with spermatozoa cryopreserved before the initiation of anticancer treatments. Until recently, none of the research reported an increased rate of congenital abnormalities or malignancies in children born from treated patients with subsequent spontaneous conceptions [52]. In a large retrospective cohort analysis of validated cases of congenital anomalies among 4,699 children of 1,128 male and 1,627 female cancer survivors an anomaly prevalence of 2.7 % has been found in the offspring of cancer survivors, similar to the prevalence in the general population of the US [56]. Conceptions from ART treatments were excluded from the study as the use of donor could not be determined [56]. For children whose fathers were exposed to radiation or alkylating agents versus neither, the prevalence of anomalies was 1.9 % versus 1.7 % (*p* = 0.79). Testicular radiation dose (mean 0.48 Gray) was not related to the risk of congenital anomalies (odds ratio [OR] = 1.01; 95 % 0.36-2.83 for 0.50+ Gray). Treatment with alkylating agents was not significantly associated with anomalies in the children of male survivors [56].

Conflicting results were demonstrated in a cohort study from Danish and Swedish registries, regarding 8,670 babies with a paternal history of cancer: a total of 8,162 children were conceived naturally and 508 were conceived using ART (i.e. IVF or ICSI) [57]. Overall, the offspring of male cancer survivors were more likely to have major congenital abnormalities than those of fathers without cancer (RR = 1.17; 95 % CI 1.05-1.31), with some differences between the different types of cancer. Although not significant, a link between paternal history of cancer and risk of major congenital abnormalities was suggested in children born within 2 years of their father’s cancer diagnosis (RR = 1.27; 95 % CI 0.89-1.80) [57]. The association between paternal history of cancer and risk of congenital abnormalities was not modified by the mode of conception, natural conception (RR = 1.17; 95 % CI 1.04-1.31) or ART (RR = 1.22; 95 % CI 0.80-1.87). Out of the 205 children conceived through ART with available information, 137 (55 %) were conceived using fresh post-treatment semen and 68 (27 %) using sperm cryopreserved before treatment initiation. The same prevalence of major congenital abnormalities were observed in children conceived using fresh post-treatment semen (4.4 %) or those born using cryopreserved pre-treatment spermatozoa (4.4 %) [57]. Although limited by small numbers, this finding suggests the possibility that the increase in abnormalities could be due to some factors unrelated to anticancer therapies.

The efficacy of ART in patients treated for cancer seems to be good. In a comparative study of male cancer survivors treated with ICSI using cryopreserved sperm, which were compared to non-cancer infertile males that had previously frozen sperm, the live birth rate using ART was similar in the two groups [58].

To our knowledge, only two studies have been published describing the offspring of patients undergoing ART conceptions with the use of fresh ejaculated or testicular semen after treatment [59, 60]. Ping and colleagues described the fertility outcome in 117 testicular cancer survivors [59]. Out of 21 patients who banked their semen before starting chemotherapy, 19 achieved conception by ART (11 patients used fresh semen and 8 used cryopreserved semen) [59]. A total of 37 healthy babies were conceived with no congenital malformations or childhood malignancies [59]. Recently, a large US experience reported the successful treatment of post-chemotherapy azoospermia with the use of microsurgical testicular sperm extraction: a total of 73 post-chemotherapy azoospermic patients who underwent ART were included [60]. Sperm was retrieved in 37 % of patients; fertilization rate by ICSI (per injected oocyte) was 57.1 %, with a reported clinical pregnancy rate of 50 % and an overall live birth rate of 42 %. A total of 15 deliveries were described, with 5 twin births. The 20 children were healthy with no apparent abnormalities; no details on obstetric outcome were reported [60].

In a recent large population-based study on the outcomes of progeny born after ART in the general population, there was no increase in the overall risk of cancer among British children born after assisted conception during the 17-year study period [61]. Increased risks of hepatoblastoma and rhabdomyosarcoma were detected, but the absolute risks were small [61].

##### Recommendation 5

Paucity of data is available on fatherhood after cancer. Although most of the published data are reassuring, some recent conflicting results suggest a potential increased risk of birth defects particularly among the children born closer to a paternal cancer diagnosis, and caution should be taken in counseling these patients (V, B for discussion with patients); data on children conceived after ART are too scarce to draw any conclusion although in the general population, available evidence for the outcomes of progeny after ART suggests safety of the techniques themselves (V, B for discussion with patients).

#### Is it safe to perform a controlled ovarian stimulation (COS) in female cancer patients?

Different protocols with different preparations and dosages, are available in standard ART for oocyte or embryo cryopreservation [62]. Two safety issues should be considered for COS in cancer patients: a possible delay in cancer treatment initiation (the 2-week duration of standard protocols), and a possible negative impact of ovarian stimulation on the prognosis of patients with hormone-responsive tumors in particular.

For many diseases, delays of 2 weeks prior to anti-neoplastic therapy are not possible (e.g. acute leukemia). However, even in the setting of adjuvant therapy, concerns arise regarding timing of therapy. In early stage breast cancer, evidence suggests that the earlier adjuvant chemotherapy is administered, in general, the better patients’ outcome is obtained [63]. Thus, efforts have been made to limit delays to preserve fertility in such settings. In recent years, random stimulation protocols inducing luteolysis have been adopted to allow to start COS practically anytime during the menstrual cycle without having to wait until the follicular phase [64], but even these protocols require at least 2 weeks of treatment, during which time cancer therapy must be delayed. Preliminary experiences with these “random-start protocols” showed promising results in terms of oocyte recovery and maturity rate (Table 4) [65–73].

**Table 4:** Published experiences with “random-start protocols”

Author	No. patients	No. of retrieved oocytes	No. of cryopreserved oocytes/embryos	Maturation rate (%)	Fertilization rate (%)
Von Wolff et al. [65]	12	10.0 ± 5.7	NR	80.4	75.6
Michaan et al. [66]	22	8.8 ± 6.0	5.4 ± 4.5	NR	NR
Bedoschi et al. [67]	2	12	7	70.0	83.3
Sonmezer et al. [68]	3	9 - 17	7 - 10	58.8 – 77.7	69.2 – 87.5
Maman et al. [69]	5	12.8 ± 8.4	6.4 ± 6.6	48.6 ± 18.3	69.2 ± 47.4
Nayak et al. [70]	4	6 - 30	5 - 20	NR	93.3 – 100
Cakmak H et al. [71]	35	9.9 (7.7 – 12.7)	NR	67 (59 – 76)	87 (72–100)
Buendgen NK et al. [72]	10	8.8 (SD: 5.1)	NR	NR	63.6 (SD: 32.9)
Keskin U et al. [73]	3	4 - 16	3 - 9	NR	75

*NR* not reported, *SD* standard deviation

The risk of ovarian hyperstimulation syndrome (OHSS), a severe complication of COS, should be taken into account, particularly in young patients with high ovarian reserve. In these cases, triggering ovulation with LHRHa instead of human chorionic gonadotropin (HCG) has been proven to significantly reduce the occurrence of OHSS, a complication that may also further delay the start of anticancer treatments [74].

The short-term exposure to high estrogen levels due to the COS is an important safety concern in patients with breast cancer. For this reason alternative protocols for ovarian stimulation with the use of tamoxifen [75] or letrozole [76] have been developed. In these relatively small studies, no negative consequence on the quality of the oocytes and embryos collected has been observed [75, 77] and pregnancy rates have been similar to those expected in a noncancer population undergoing IVF [78]. The largest prospective analysis with the use of cryopreservation strategies in breast cancer patients reported the safety of a COS with the use of letrozole in 79 women who were compared with 136 patients not undergoing any fertility-preserving procedures serving as controls [79]. After a median follow up of 23.4 months post-chemotherapy, no difference in relapse-free survival was observed among the two groups (HR = 0.56; 95 % CI 0.17-1.9; *P* = 0.36) [79]. Recently, updated results of the study, including a larger sample size (338 patients, 120 women undergoing COS and 218 controls) with a longer median follow up (4.9 years), confirmed the safety of the procedure [80]. Safety and feasibility of performing two consecutive COS with the use of letrozole to increase the oocyte/embryo yield for fertility preservation has also been reported [81]. However, further research in this filed is needed on larger series to confirm these results.

To avoid the need for COS, cryopreservation of immature oocyte or of oocytes matured in vitro are under clinical development [82, 83]. With these techniques, oocytes are collected without hormonal stimulation or with a short stimulation lasting 3–5 days; the immature oocytes collected can be then cryopreserved after maturation in vitro or cryopreserved at the immature stage and then matured in vitro after thaw before insemination. So far, only one child was born in a cancer patient in whom the immature oocytes were collected prior to gonadotoxic treatment [84]. These strategies should be considered still experimental.

To date, only one study has reported the safety of performing ART following anticancer treatments for breast cancer treatment [85]. The authors evaluated the survival outcomes of a cohort of 198 patients who became pregnant after breast cancer diagnosis and treatment of whom 25 women underwent ART resulting in 36 pregnancies [85]. A total of 37 ART cycles were performed: 13 oocyte donation, 13 ovarian stimulation for IVF, and 11 ovulation induction. Patients who underwent ART tended to have more favorable characteristics (e.g. higher percentage of estrogen receptor positive disease, low grade tumor and node negative status) [85]. At a median follow up of 50 months, no difference in DFS and overall survival has been observed between patients with spontaneous pregnancies and those who underwent ART procedures [85]. However, more data are needed to confirm the safety of performing a COS in patients with prior diagnosis and treatment of breast cancer.

##### Recommendation 6

The current limited data suggest the safety of a COS in cancer patients (III, B). “Random-start protocols” can be employed to avoid delays in anticancer treatment initiation (III, B). LHRHa ovulation triggering should be adopted in patients at moderate-high risk for OHSS (I, A). Letrozole (or tamoxifen) should be incorporated in the protocol for COS in cancer patients with hormone-responsive tumors (III, B).

#### Which method for cryopreservation of embryos or oocytes should be used and what should we expect from the use of this strategy in female cancer patients?

Embryo cryopreservation and oocyte cryopreservation are standard strategies for fertility preservation in female cancer patients [10, 11]. In countries where embryo freezing is prohibited by law (e.g. Italy), oocyte cryostorage is the only applicable non-experimental technology.

Two different methods for embryo or oocyte cryopreservation are currently available: slow freezing and vitrification [86]. Slow freezing procedure has been the first procedure to be developed: with this method, eggs are gradually frozen with low concentration of cryoprotectant agents, thus minimizing both structural damages and intracellular formation of ice [87]. Vitrification uses higher concentrations of cryoprotectants and an ultra-quick cooling is performed to avoid toxicity and to reduce the transitional stage; egg survival and fertilization rates with vitrification are expected to be higher, since the structural damage due to the formation of ice crystals is avoided [88]. In a recent Cochrane meta-analysis of two studies, vitrification showed a higher clinical pregnancy rate than slow freezing (RR = 3.86, 95 % CI 1.63-9.11) and better results in terms of oocyte survival rate, fertilization rate and embryo quality [89]. However, no information on live birth rates was reported. In experienced laboratories, results with vitrification may be similar to those obtained with fresh eggs but success rates are still highly operator-dependent [90]. Irrespective of which method is used, the success rates are best in women under 36 years, although success has been reported up to age 44 with vitrification [91].

In Italy, due to the legal restrictions limiting the use of embryo cryopreservation, an increasing trend toward the use of oocyte freezing has been shown since 2004. Using data from the Italian Registry of ART in the period 2007–2011, Levi Setti and colleagues showed an increasing use of vitrification in infertile couples and by 2010 vitrification became the most applied technique [92]. The success of oocyte cryopreservation with slow freezing and vitrification were then retrospectively compared, confirming a statistically significant higher performance of vitrification than slow freezing, although not as high as with fresh cycles [92].

Most of the available data on pregnancies obtained with thawing of embryos or oocytes derive from the infertile non-oncologic population. Pregnancy rate after embryo thawing is strongly dependent on age, ranging from over 40 % in women younger than 35 years to less than 20 % in women over 40 years [93]. Similar results in experienced hands have been shown after oocyte cryopreservation [94, 95]. The number of oocytes or embryos stored is another factor that strongly impacts on the outcomes.

Cancer patients might have a weaker response to COS [96]: a retrospective observational study reported a lower number of retrieved oocytes in cancer patients as compared to a historical control group of age-matched women who underwent ART [97].

As a matter of fact, many issues should be considered when analyzing ovarian response to COS in cancer patients, such as the particular protocols used (i.e. “random-start protocols” to avoid the delay in treatment initiation, or the use of tamoxifen or letrozole in patients with hormone sensitive diseases to reduce the risk of the exposure to the high estradiol levels during COS) and/or the presence of a possible underlying reduced ovarian reserve (e.g. patients with BRCA 1–2 mutations or due to concurrent illness) [98, 99].

To increase the oocyte yield for fertility preservation, it has been proposed to perform two consecutive ovarian stimulation cycles [81]. However, this approach is not feasible in many cancer settings due to the delay it would necessitate in starting treatment.

Very limited data are available on pregnancy rates with thawed oocytes or embryos in cancer patients. To date, a total of 8 deliveries have been reported after oocyte warming in the oncologic population [100]. Recently, out of 357 patients who had their oocytes cryopreserved after cancer diagnosis, 11 cancer survivors (8 with breast cancer, 1 with Hodgkin lymphoma, 1 with endometrial adenocarcinoma, and 1 with thyroid cancer) returned for ART [101]. In this cohort, a total of 4 pregnancies were achieved and delivered at term after warming vitrified oocytes with no major or minor malformations; the delivery rate per cycle was calculated to be 36.6 % [101]. Even more recently, Oktay and colleagues provided the pregnancy and fertility preservation outcomes in a cohort of 131 breast cancer patients who had previously undergone COH with letrozole for embryo cryopreservation [78]. A total of 33 women underwent 40 attempts to transfer embryos obtaining 18 pregnancies and 25 live births [78]. Pregnancy rates were comparable to those expected in the general noncancer population undergoing IVF [78].

##### Recommendation 7

Embryo and oocyte cryopreservation are standard options for fertility preservation (III, B). Vitrification showed a better performance than slow freezing (II, B). During oncofertility counseling, patients should be aware that data on the success of these strategies derive from infertile women in general and that a different ovarian response to stimulation might be expected in cancer patients (IV, B).

#### Is there any best candidate for ovarian tissue cryopreservation?

Ovarian tissue cryopreservation has been proven to be an effective, yet still experimental, technique to preserve fertility in patients undergoing gonadotoxic therapies [10, 11]. Following re-implantation of ovarian tissue, a successful recovery of ovarian function is expected in almost all cases within 3 to 6 months, with possible sustained longevity of function of the transplanted tissue [102, 103]. To date, a total of 40 live births have been reported in cancer patients after transplantation of frozen/thawed ovarian tissue [104]. It is basically impossible to calculate a precise pregnancy rate for transplantation of cryopreserved ovarian tissue until a large cohort of patients have had all their tissue transplanted [105]. Combining 80 cases from 4 fertility centers, the pregnancy rate (i.e. ratio between the number of women who conceived and the number of transplanted women) with the use of this technique was 25 % [106–109]. Overall, the pregnancy rate with the use of cryopreservation of ovarian tissue seems to be increasing [105].

Although ovarian tissue cryopreservation is still an experimental strategy, it might be proposed to selected patients. In particular, this method is the only available option to preserve fertility in prepubertal girls who are candidates to gonadotoxic therapies [40].

A major advantage of this technique over oocyte or embryo cryopreservation is that only few days are required for its application: tissue retrieval can be performed by laparoscopy, which can be planned shortly after cancer diagnosis, or during a laparotomy if needed for oncologic therapies. This technique can be performed at any time of the menstrual cycle and does not require hormonal stimulation. For these reasons, cryopreservation of ovarian tissue can be proposed to selected patients who cannot delay the initiation of anticancer treatments [110].

Another promising indication of ovarian cortex cryopreservation is the situation in which patients have already received chemotherapy. Mature or immature egg collection is not recommended in patients who have received recent chemotherapy, owing to possible decreased or no response to ovarian stimulation, genetically abnormal oocytes, and deleterious effects on reproductive outcome (i.e. high abortion and malformation rates), as suggested by animal studies [111, 112]. On the contrary, the collection of ovarian tissue may be successfully performed after a few cycles of chemotherapy due to the fact that a substantial number of primordial follicles will still survive and be present in cortical tissue [113].

The success of ovarian tissue cryopreservation is strongly dependent upon the patient’s ovarian reserve. For this reason, patients over 40 years or with reduced ovarian reserve are not good candidates [114]. Furthermore, a potential risk of reintroducing malignant cells when the tissue is re-implanted should be considered [115–117]. Therefore, patients diagnosed with cancer with a high risk of malignant contamination to the ovaries (e.g. aggressive hematologic malignancies) and with no reliable molecular markers for a pre-transplantation examination may be not eligible for ovarian tissue auto-transplantation [118]. Finally, it should be specified that the possibility to reduce ovarian reserve with ovarian cortex biopsy has not been well studied to date.

##### Recommendation 8

The best candidates for ovarian tissue cryopreservation are prepubertal girls (III, A). The technique may also be proposed to patients scheduled for treatments with a high risk of premature ovarian insufficiency who cannot delay anticancer treatments or who have already received chemotherapy, or with contraindications to COS (III, B). Patients with cancer with a high risk of malignant contamination to the ovaries (e.g. aggressive hematologic malignancies) should not be considered eligible for ovarian tissue auto-transplantation (V, B).

#### Should ovarian cortex tissue banking be performed locally or should it be centralized?

Embryo and oocyte cryostorage is commonly applied in several fertility units as part of infertility treatments, whereas ovarian cortex cryopreservation is applied only as a technique for fertility preservation in patients at risk of gonadic exhaustion. Moreover, the effective assessment of a program of ovarian tissue cryopreservation requires time: in fact, many years may pass before a sample is thawed and transplanted. Due to the relatively low number of procedures requested and the difficulty in evaluating survival of primordial follicles, the technique of ovarian cortex cryopreservation should be concentrated to few centers with the appropriate expertise [10, 11].

Animal experiments showed that the conservation of ovarian tissue at 4 °C for up to 18 hours causes no morphological damage to preantral follicles; on the contrary, the storage of ovarian tissue at 20 °C for 18 hours significantly reduces the percentage of morphologically normal follicles [119]. Several reports have demonstrated the feasibility of harvesting the ovarian cortex in one site and then transporting it to another site to be frozen. In specific cooling devices, primordial follicles can survive to transportation for up to 4–6 hours prior to cryopreservation [120, 121]. Pregnancies after ovarian tissue cryostorage following a transportation for more than 20 hours have also been reported [122].

Centers that perform cryopreservation, storage and transplantations of ovarian tissue require a connection with highly specialized laboratories for detecting traces of malignant cells in the sample at both pathologic and molecular levels before the grafting. Optimization of freezing methods and cancer cell detection techniques are other two valid reasons to concentrate the application of an “experimental technique” in a few referral centers. On the other hand it would be preferable to organize the harvesting of the tissue locally to ensure the access to this technique to all the eligible patients. Even though some skills are required to perform an ovarian cortex biopsy suitable for cryopreservation, any gynecological or fertility unit may easily acquire them.

##### Recommendation 9

In order to optimize the procedure in terms of both patient management and cost-effectiveness, the harvesting of the tissue can be performed locally but subsequent sample freezing and storage centralized (III, B). A well-organized network between fertility units is required (III, B).

#### Should ovarian suppression with LHRHa be proposed as a reliable strategy to preserve ovarian function and fertility during chemotherapy?

Ovarian suppression with the use of LHRHa during chemotherapy is an attractive option to preserve gonadal function and fertility given the wide availability of such agents and the advantage of causing no delay in the initiation of anticancer therapies [123]. A total of 10 randomized studies in patients with breast cancer [124–133], 2 in women with lymphoma [134, 135] and 1 in ovarian cancer patients [136] have been conducted to evaluate the efficacy of this strategy: overall, these trials reported conflicting results. Heterogeneous target population and differences in the patient populations, chemotherapy regimens used, duration of follow up and study end-points are the major limitations in comparing these studies [137]. A total of 12 meta-analyses have attempted to summarize the data from all these trials [138–149]: the majority of them showed a potential efficacy of the technique in reducing the risk of premature ovarian failure, especially in breast cancer patients.

However, despite this research effort, ovarian suppression with LHRHa during chemotherapy is still considered an experimental strategy to preserve fertility by some international guidelines due to both the uncertainty regarding the efficacy of this strategy and the absence of data on pregnancies and long-term ovarian function [10, 11].

Recently, two large randomized trials evaluating the efficacy of ovarian suppression with LHRHa during chemotherapy in breast cancer patients have reported long-term outcome results [133, 150]. Of note is that in the POEMS-SWOG S0230 trial only patients with endocrine-insensitive disease were enrolled, while the majority of women in the PROMISE-GIM6 study had hormone receptor-positive breast cancer. Both trials reported a statistically significant reduction in the incidence of chemotherapy-induced POF in patients receiving LHRHa, one year after the end of chemotherapy in the PROMISE-GIM6 study (OR = 0.28; *P* < 0.001) [127] and two years after the end of chemotherapy in the POEMS-SWOG S0230 trial (OR = 0.30; *P* = 0.04) [133]. An increased pregnancy rate was reported by both the POEMS-SWOG S0230 (OR = 2.45; *P* = 0.03) [133] and the PROMISE-GIM6 (age-adjusted HR = 2.40; *P* = 0.20) studies [150]. Furthermore, an increased probability for menstrual resumption at longer follow-up (age-adjusted HR = 1.48; *P* = 0.006) was shown in the PROMISE-GIM6 study for patients in the LHRHa arm [150]. A recent meta-analysis including all the randomized trials conducted in breast cancer patients confirmed the efficacy of temporary ovarian suppression with LHRHa during chemotherapy in reducing the risk of treatment-related premature ovarian failure (OR = 0.36; *P* < 0.001) and increasing the pregnancy rate (OR = 1.83; *P* = 0.041) [149].

Recently, the 2015 St. Gallen International Expert Consensus panel and the National Comprehensive Cancer Network (NCCN) guidelines have been updated to acknowledge the use of LHRHa in preventing chemotherapy-induced ovarian failure of hormone receptor negative breast cancer patients [151, 152]. An individual patient data meta-analysis of randomized studies conducted in breast cancer patients is ongoing with the aim to better elucidate the efficacy of ovarian suppression with LHRHa as a strategy to protect both ovarian function and fertility (PROSPERO registration number: CRD42014015638) [153].

Ovarian suppression with LHRHa during chemotherapy can be used in combination with other preservation techniques including cryopreservation strategies, thus increasing the chance of both fertility and gonadal function preservation after anti-cancer systemic therapies.

##### Recommendation 10

Ovarian suppression with the use of LHRHa during chemotherapy should be considered a reliable strategy to preserve ovarian function and fertility, at least in breast cancer patients, given the availability of new data suggesting both the safety and the efficacy of the procedure have become available (I, A)*.

(*CYA, HAA, GBLS and WHW disagree with this statement, considering the strategy still experimental).

## Conclusions

Over the last several years, thanks to improvements in the prognoses of cancer patients, increasing attention has been given to survivors’ fertility issues. International guidelines on this topic have been recently updated and published [10, 11]. However, since then, new data have become available, and several issues in this field remain controversial. On the basis of the discussion during the meeting, the expert panel has drafted a total of 10 recommendations (Table 5).

**Table 5:** The 10 recommendations drafted by the expert panel

Recommendations
1) Ovarian stimulating drugs with standard treatment protocols may be administered in subfertile/infertile women without increasing the risk of developing breast cancer (III, B). The long-term use of clomiphene outside the current limited indications (i.e. first-line therapy of WHO Group II anovulatory infertility) should be discouraged because of a possible increase in breast cancer risk (III, B).
2) Pregnancy in cancer survivors, after adequate treatment and follow up, should not be discouraged, including among patients with endocrine-sensitive breast cancer (III, A).
3) All patients with potential interest in keeping their fertility should be referred to fertility unit for adequate determination of risk of infertility, chances of future conception and how to proactively preserve it (V, A). However, some cancer patients will not require the help of a fertility clinic after cancer treatment (V, B). Since several patient- and treatment-related factors are associated with the risk of developing infertility, the oncofertility counseling should be tailored to the individual patient (V, A).
4) In men, sperm cryopreservation is an easily accessible and widely available option in more than 95 % of patients and should be encouraged for those who want to preserve fertility (III, A). On the contrary, from 2 % to 65 % of women undergo one of the available cryopreservation options: oncologists should discuss with them the fertility issues and secure proper counseling in appropriate centers prior to cancer treatment (IV, A).
5) Paucity of data is available on fatherhood after cancer. Although most of the published data are reassuring, some recent conflicting results suggest a potential increased risk of birth defects particularly among the children born closer to a paternal cancer diagnosis, and caution should be taken in counseling these patients (V, B for discussion with patients); data on children conceived after ART are too scarce to draw any conclusion although in the general population, available evidence for the outcomes of progeny after ART suggests safety of the techniques themselves (V, B for discussion with patients).
6) The current limited data suggest the safety of a COS in cancer patients (III, B). “Random-start protocols” can be employed to avoid delays in anticancer treatment initiation (III, B). LHRHa ovulation triggering should be adopted in patients at moderate-high risk for OHSS (I, A). Letrozole (or tamoxifen) should be incorporated in the protocol for COS in cancer patients with hormone-responsive tumors (III, B).
7) Embryo and oocyte cryopreservation are standard options for fertility preservation (III, B). Vitrification showed a better performance than slow freezing (II, B). During oncofertility counseling, patients should be aware that data on the success of these strategies derive from infertile women in general and that a different ovarian response to stimulation might be expected in cancer patients (IV, B).
8) The best candidates for ovarian tissue cryopreservation are prepubertal girls (III, A). The technique may also be proposed to patients scheduled for treatments with a high risk of premature ovarian insufficiency who cannot delay anticancer treatments or who have already received chemotherapy, or with contraindications to COS (III, B). Patients with cancer with a high risk of malignant contamination to the ovaries (e.g. aggressive hematologic malignancies) should not be considered eligible for ovarian tissue auto-transplantation (V, B).
9) In order to optimize the procedure in terms of both patient management and cost-effectiveness, the harvesting of the tissue can be performed locally but subsequent sample freezing and storage centralized (III, B). A well-organized network between fertility units is required (III, B).
10) Ovarian suppression with the use of LHRHa during chemotherapy should be considered a reliable strategy to preserve ovarian function and fertility, at least in breast cancer patients, given the availability of new data suggesting both the safety and the efficacy of the procedure have become available (I, A)*. (*CYA, HAA, GBLS and WHW disagree with this statement, considering the strategy still experimental).

Although there is a great interest in this field, due to the lack of large prospective cohort studies and randomized studies on these topics, the level of evidence is not higher than 3 for most of the recommendations highlighting the need of further research efforts. Several registries and prospective studies are ongoing to evaluate feasibility, safety and efficacy of fertility preserving strategies in cancer patients and encouraging patient participation in these studies is crucial to acquire more robust conclusions.

## Abbreviations

ASCO: American Society of Clinical Oncology
ESMO: European Society for Medical Oncology
US: United States
RR: relative risk
CI: confidence intervals
HRT: hormone-replacement therapy
SRR: summary relative risk
IVF: in vitro fertilization
HR: hazard ratio
PRR: pooled relative risk
DFS: disease-free survival
ABVD: doxorubicin/bleomycin/vinblastine/dacarbazine
LHRHa: luteinizing hormone-releasing hormone analogs
ART: assisted reproduction technologies
ICSI: intracytoplasmatic sperm injection
OR: odds ratio
COS: controlled ovarian stimulation
OHSS: ovarian hyperstimulation syndrome
HCG: human chorionic gonadotropin
NCCN: National Comprehensive Cancer Network
